# A Brief Account of a Fever Which Prevailed in the Mountain District of Tennessee, in the Fall and Winter of 1847-’8

**Published:** 1849-03

**Authors:** M. Y. Brockett

**Affiliations:** Spencer, Tennessee


Art. III.—*A brief account of a Fever which prevailed in the mountain district of Tennessee, in the Fall and Winter of* 1847–'8. By Dr. M. Y. Brockett, of Spencer, Tennessee.

The region of country in which the fever of which I propose to give a short account prevailed, lies to the east of Sparta, and is watered by the Cany fork of Cumberland river. The country is mountainous and free from swamps or collections of stagnant water, and the streams are bold and clear. Sandstone and limestone both enter into the geology of the country, so that we have both free stone and limestone water. No local cause of disease appears anywhere upon the surface.

The summer of 1847, and the first months of autumn were unusually wet and warm. Few thunder storms occurred, but not unfrequently the rains continued for eight or ten days, in consequence of which the streams were swollen to an uncommon degree.

The fever which succeeded this extraordinary weather was not confined to the valleys and margins of streams, but appeared also on the tops of the mountains. The type was typhoid, as will appear from the history of the following case:

Miss M., æt. about 14 years, was seized with the disease on the 2d of December, and on the 4th I found her laboring under the following symptoms: severe pain in the head; general debility; listlessness, and dullness of intellectual faculties; intolerance of light; anorexia; constipation; nausea. Her pulse was quick, but soft and yielding; surface not preternaturally hot; great thirst; sleeplessness; tongue coated with a brown fur. In this state she remained until the fifth day, the symptoms appearing rather to progress. On the sixth day they were decidedly aggravated. The tongue became black; sordes formed on the teeth, and these phenomena were attended with low muttering delirium, and extreme restlessness. Bowels still constipated; retention or suppression of urine; eyes suffused, and with a wild expression. This case presents a fair type of the disease.

In the treatment of this fever I did not have recourse to the lancet, for the reason that one of the first cases appeared to me sensibly injured by the abstraction of blood. It fell into the hands of a physician of Sparta who bled the patient; and this was one of the few fatal cases that occurred. The symptoms did not seem to justify bloodletting. The strength of the patients was not equal to it. They were exhausted from the onset of the disease. In no case were the effects of venesection satisfactory.

The first medicine I gave, when called to a case, was a dose of calomel and jalap. If they did not produce a copious evacuation in the course of eight hours, they were followed by castor oil or senna. The stools were generally black, sometimes bloody; and yet the latter symptom was not serious, for uniformly when it made its appearance I was able to prononunce a favorable prognosis. The cerebral excitement was allayed by the alvine discharges, and when they were attended by pain in the bowels, and tenesmus, I had recourse to small doses of calomel, ipecacuan, and opium, alternated with olive oil, spirits of turpentine, and tinct. opium, and these remedies never failed to relieve the intestinal uneasiness, and allay the febrile excitement. Convalescence was speedily established, the disease appearing to be cut short by the treatment.

The following are the minutes of a case of the fever treated by my partner, Dr. D. G. Hughes:

Mrs. C. was taken, about the 12th of Oct., with headache and a feeling of general debility.

13th. Dry, hot skin; thirst; constipated bowels, and all the symptoms of a well marked case of fever.

14th. To the other symptoms, nausea and frequent efforts at vomiting have been added. Calomel and rhubarb were given, and their action promoted by an enema of senna tea. Great relief of the head followed their action upon the bowels; but in place of the cerebral symptoms, griping and other indications of intestinal irritation appeared. For these, I prescribed olive oil, spirits of turpentine, and tinct. opium.

15th. Symptoms are unchanged; bowels loose, the discharges being green and curdled; breath fetid; cough, and free expectoration of mucus. Prescribed hive syrup, syrup of squills, and liquorice, which excited vomiting.

16th. Cephalic symptoms renewed. Blister to the nape of the neck.

17th. Retention of urine; symptoms otherwise the same. Treatment—calomel, ipecacuan, and morphia in minute doses.

18th. Tongue black and chapped, teeth covered with sordes. Wine was given, but aggravated the symptoms. Continue treatment.

20th. Patient convalescent, having slept soundly and awoke refreshed.

I do not present these as cases of true typhoid fever, but as cases of bilious fever of a typhoid character. They exhibited many of the marks of the former disease, but ran their course more rapidly than it is understood to do. The interesting feature regarding it, if interest it possesses at all, is that it appeared in a mountainous region of country, which, from its aspect, would seem to be free from all sources of malaria, and followed a season which, by its excessive humidity, might generate the poison of fever in any situation where there was the requisite amount of heat and vegetable matter.

Before closing this communication I will detail the particulars of a fatal case of exanthematous disease which interested me a good deal in its progress, and possibly may have interest for the readers of the Journal.

I was called in September last to see a child, 5 years of age, laboring under what I regarded as a form of erysipelas. Its lower extremities were covered with a papular eruption, attended by intolerable itching. I used antiphlogistic remedies, under which the affection seemed to be giving way, and after a few days I dismissed my little patient as convalescent. I heard nothing from him for three weeks, when I was again called, and found that the eruption had spread extensively over the abdomen. Large indurated ulcers in a short time appeared on every part of his body, under the exhausting effects of which he rapidly sunk.

Spencer, January 30th, 1849.

